# Forgone healthcare for medically vulnerable groups during the pandemic era: experiences of family caregivers of young adults with substance use disorders in Zambia

**DOI:** 10.3389/fpubh.2024.1250608

**Published:** 2024-03-08

**Authors:** Ireen Manase Kabembo

**Affiliations:** ^1^Department of Sociology and Social Policy, Lingnan University, Hong Kong, Hong Kong SAR, China; ^2^Department of Social Work and Sociology, University of Zambia, Lusaka, Zambia

**Keywords:** forgone healthcare, COVID-19 pandemic, medically vulnerable groups, family caregivers, young adults, substance use disorders, Zambia

## Abstract

**Introduction:**

Scholars worldwide have defined the COVID-19 pandemic as a mass-disabling event of our time. The situation is grave for families experiencing financial hurdles while caring for young adults in recovery from addiction problems.

**Methods:**

Using semi-structured interviews with 30 purposively selected family caregivers (FCGs) of young adults with substance use disorders (SUDs) in Lusaka, Zambia, this study reveals several factors influencing forgone healthcare for this medically vulnerable group.

**Results:**

Financial challenges and huge out-of-pocket bills; caregivers’ perceived far-fetched recovery of the young adult; the cost of medication and transportation; the young adult’s little perceived need for healthcare service use, their runaway and treatment elusive tendencies; caregiver concerns about contracting the virus, and the stigma associated with it; and a fragmented child and adolescent mental health system influenced forgone healthcare. The young adults were often unavailable for days and months, posing challenges to the continuity of care. Despite caregivers’ acknowledgment of the availability of healthcare professionals, young adults with problematic substance use had limited access to SUD recovery services, resulting in adverse health outcomes. Results also show that most family caregivers encountered challenges in accessing and purchasing psychotropic medications, which were difficult to find during the lockdowns. Some family caregivers lost their sources of income by being laid off from work due to the pandemic and skipping work to attend to caregiving responsibilities. Most of those in self-employment had to close their business and stay home to look after their youth. Several caregivers kept their youth at home because they failed to access private residential SUD recovery services. Family caregivers mostly relied on outpatient public health services, alternative medicine from traditional healers, and faith-based healing, all of which some young adults rarely accessed because of their problematic behaviors of escaping healthcare.

**Conclusion:**

These identifiable risk factors, and their detrimental consequences highlight the need for interventions to improve healthcare access for this vulnerable population. Supporting FCGs of addicted young adults is crucial in ensuring the well-being of both the caregivers and care recipients. Further research is warranted to explore potential solutions, such as peer support programs, policy changes, and education initiatives for carers and recipients in the (post) pandemic era.

## Introduction

Family caregivers’ lived experiences in caring for young adults with substance use disorders remain a critical yet often overlooked aspect of addiction recovery. These caregivers face many challenges while supporting their loved ones, navigating the complexities of addiction, and striving to maintain their wellbeing (1, 2).

Although research on forgone healthcare is widespread in developed countries and mainly focuses on individuals with health problems, there is a pervasive void in research on forgone healthcare in Low- and Middle-Income Countries. According to the extant literature, “The concept of foregone care focuses on conditions under which people chose not to, or are not able to, use health services despite perceiving a need for those services” [(3), p. 775]. In showing the paucity of studies on forgone healthcare in LMICs during the COVID-19 pandemic, Kakietek et al. (3) argued that “despite the greater vulnerability of households and fragility of the health systems, there are no published studies on the prevalence of foregone healthcare during the pandemic in LMICs” [(3), p. 772]. To fill this profound gap in research, this paper reports on the lived experiences of forgone healthcare among family carers of adolescents and young adults grappling with addiction during the COVID-19 pandemic in Zambia and argues that caregiving for young adults with substance use disorders is a challenging task, involving psychosocial, physical, and financial strains. This is because young adults with SUDs are among the medically vulnerable groups in society. Weitzman et al. (4) define medically vulnerable groups as those with chronic illnesses, for example chronically ill youth [(4), p. 450] who have an increased risk of developing severe illness if they contract a particular disease or condition. Due to the chronicity of their illnesses, these individuals are more susceptible to complications and adverse outcomes. SUDs are chronic illnesses which have been defined as “complex disorders that affect brain function and behavior, are characterized by impaired functioning and considerable harm to the individuals with the disorders and to society as a whole” [(5), p. 2]. Addiction scholars posit that as a medical brain condition, SUD involves the consumption of illicit substances like cocaine, cannabis, heroine or methamphetamine and legal substances like alcohol and prescription medications. Among legal drugs linked to substance use disorders, alcohol is the most prevalent (6), with affected individuals struggling to regulate their consumption (7).

According to Menon et al. (8), youth substance use problems are prevalent in Lusaka, Zambia, with around 38.5% of facility-based admissions related to alcohol use disorders whereas, 12.4% are attributable to cannabis or marijuana, cocaine, and heroin (9). Crane et al. (10) recorded a significant increase of 293% in hazardous alcohol consumption admissions between 2010 and 2014 at the country’s largest psychiatric hospital. In more recent research on the prevalence of addictive behaviors among adolescents from 73 low-and middle-income countries, de la Torre-Luque et al. (11) found that Zambian adolescents had the highest regular and problematic use of alcohol. The social acceptance and prevalence of alcohol consumption as part of Zambian traditions and celebrations, coupled with the availability of cheap and unsafe varieties of alcoholic beverages due to economic liberalization, have contributed to the high rate of alcohol consumption in the country (8, 12, 13).

Moreover, the lack of recreational facilities for young people and elevated levels of poverty and unemployment are contributing factors to the increased intake of alcohol in Zambia, as stated by the Ministry of Health (13). All these factors indicate the widespread prevalence of SUDs in Zambia, emphasizing the need for further research on the experiences of caretakers who care for young persons diagnosed with SUDs, as research on caregivers of substance-dependent individuals is nearly non-existent in Zambia. Therefore, this study fills this gap in research on addiction care in Zambia.

In addition to creating changes in addiction problems, the COVID-19 pandemic has changed how individuals access and receive medical care. The pandemic’s effects go beyond the direct impact of the virus, and it has also affected the healthcare-seeking behavior of individuals. Many individuals have been unable or unwilling to access healthcare services during the pandemic, leading to forgone healthcare. This phenomenon is a significant public health concern as it can worsen chronic conditions, delay diagnoses, and increase morbidity and mortality (14).

Several factors have contributed to the decrease in healthcare-seeking behavior during the pandemic, including the fear of contracting the virus, lockdowns, and stay-at-home orders. Financial constraints were also a significant barrier to healthcare, with many people experiencing job losses and reduced income. According to Kakietek and colleagues (3), close to 52% of respondents in Sub-Saharan Africa reported forgoing healthcare due to financial constraints as opposed to 16.7% who forwent care due to COVID-related reasons. Conversely, Menon et al. (14) found that most who had forgone healthcare had done so because they were concerned about contracting COVID-19 or burdening the healthcare system.

The consequences of forgone healthcare before, during, and after the pandemic are grave and long-term. Forgone healthcare can severely affect individuals’ health outcomes, particularly those with chronic conditions. Delayed diagnoses can lead to the progression of diseases, and lack of medical attention can lead to increased morbidity and potentially life-threatening outcomes, and lower quality of life (15, 16).

The Canadian Centre on Substance Use and Addiction (17) reports that on a global level, alcohol use has dropped by approximately 10%–15% during the pandemic. However, various sectors of society, like the unemployed, those with alcohol abuse issues, and people in precarious situations, have seen a rise in such consumption. For most young adults, the COVID-19 pandemic predicted increased substance use due to a sharp rise in depression, anxiety, stress, and boredom that came with the isolation (18). This resulted from disruptions in the daily routines of adolescents and young adults. School closures meant a breakdown in physical interaction with their peers and non-engagement in extracurricular activities that involve the participation of more than one person, such as sports.

While numerous studies have been done on the impact of the pandemic on young people’s developmental outcomes, there is a paucity of studies on young people with problematic substance abuse, particularly regarding the lived experiences of family caregivers of adolescents and young adults with problematic substance use in Sub-Saharan Africa. This has limited our understanding of forgone healthcare for this at-risk group. The present research, therefore, aims to fill this knowledge gap.

### Brief overview of the situation of COVID-19 in Zambia

On 18th March 2020, Zambia recorded its first case of COVID-19 in a couple that returned from a holiday in France. Although the cumulative number of deaths has generally been low compared to other countries, the impact of the pandemic on families of young adults with substance use disorders has been grave. As of 28 June 2023, 347,928 positive cases were confirmed, with 4,064 deaths (19).

Even before COVID-19, Zambia’s economic condition and outlook were bleak. Factors such as falling copper prices, a substantial external and internal debt burden, rising inflation, and poor management of the public sector (20) characterized the Zambian economy. Therefore, with the pandemic’s adverse effects on the economy, the Zambian government lacked the necessary resources to tackle the additional economic challenges brought on by the COVID-19 pandemic, placing an undue burden on Zambian households (21). Furthermore, Saasa and James (21) note that economically afflicted homes are at a higher risk of experiencing even more severe economic circumstances due to the pandemic because, even before COVID-19 struck, Zambian households faced various difficulties such as poverty, hunger, and joblessness. Regrettably, these problems are predicted to worsen during this outbreak and beyond, as most individuals in the informal sector depend on daily income. In Zambia, approximately 87.5% are engaged in informal sector employment (22).

Despite not implementing a total lockdown, the Zambian government took measures to curb the virus, including travel restrictions, limitations on meetings in public spaces, stay-at-home orders, and social distancing. Like many countries in the region that implemented such measures, these measures had dire consequences on Zambian businesses and families, resulting in many individuals losing their jobs and income and consequently being unable to sustain their livelihoods and fend for people in their care (21). This situation made FCGs more vulnerable to the economic downturn as most were in sectors hard-hit by the pandemic.

As Finn and Zadel (23) show in their phone survey on monitoring COVID-19 impacts on households in Zambia, individuals engaged in non-farm businesses experienced a 71% reduction in income and an 11% complete loss of income, as shown in Table 1.

**Table 1:** Change in income from four main household income sources since outbreak.

Household income source	Increased	Stayed the same	Reduced	Stopped
Farming, livestock or fishing	18%	23%	51%	8%
Non-farm business	7%	10%	71%	11%
Wage employment	2%	65%	26%	7%
Remittance from family in Zambia	8%	20%	58%	14%

Source: Finn and Zadel (23).

The massive loss of jobs in different economic sectors fueled this income reduction. For instance, the personal services sector was the second highest sector, with a 39% job loss after the tourism sector, which recorded a 71% loss of jobs among respondents in the survey by Finn and Zadel (23).

**Figure 1:**
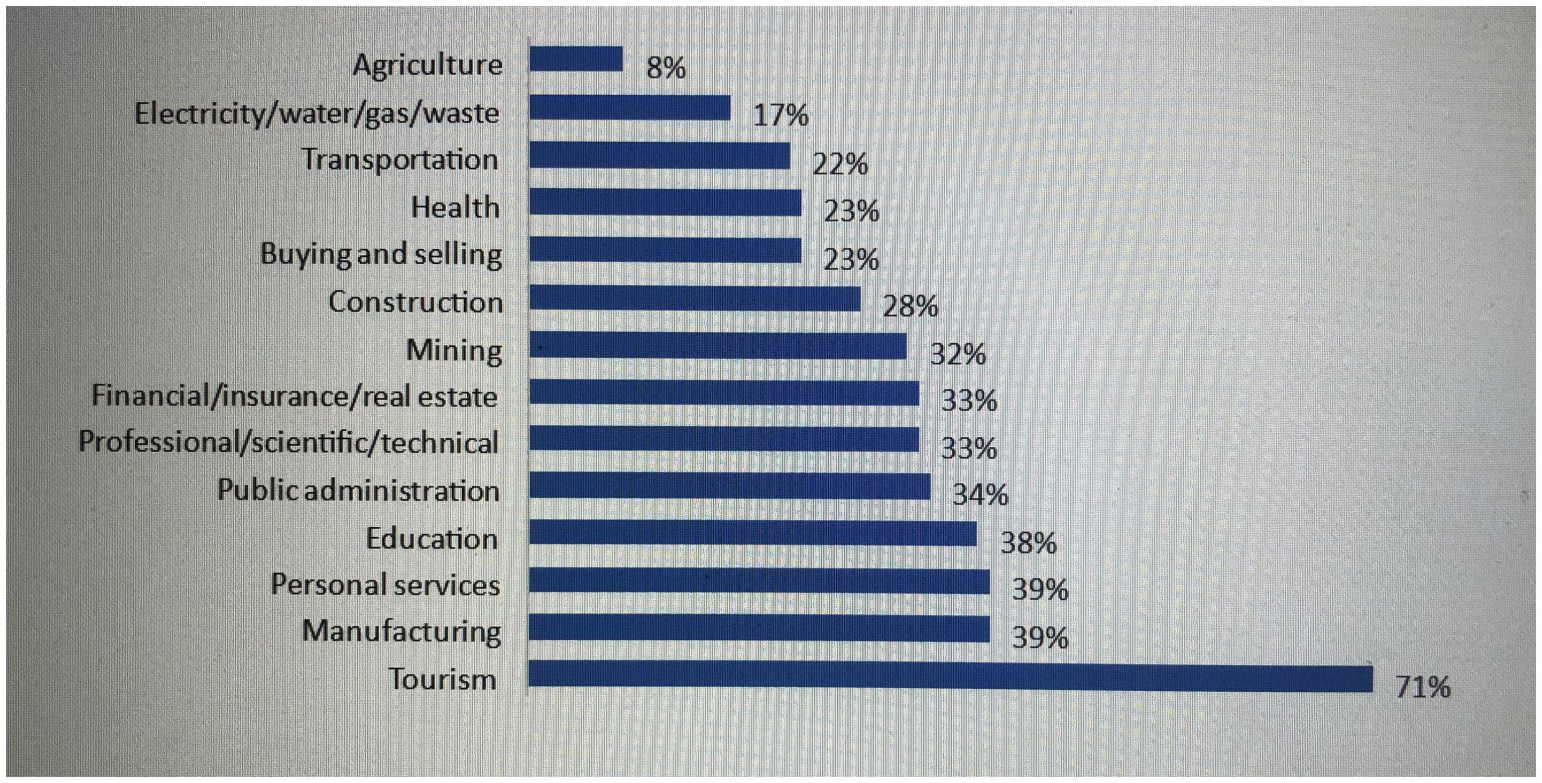
Respondent job losses by sector since outbreak began. Source: Finn and Zadel (23).

Figure 1 shows that job losses during the COVID-19 pandemic in Zambia were much higher in non-agricultural sectors, affecting many households in Lusaka that rely on personal services such as street vending, small enterprises, and domestic work.

Also, Paul and colleagues assert that “With a Gini index of 57.1, Zambia is also among the most unequal countries in the world” [(24), p. 2], with persistently high poverty levels that have stagnated at 54% for more than 10 years. Therefore, if left unabated, the pervasive inequalities entrenched in Zambian society will likely persist after this mass-disabling pandemic has receded. These underlying disparities, which affect access to resources, opportunities, and essential services, have profound implications for the wellbeing of vulnerable groups and the broader socioeconomic landscape of the country. Efforts to address these structural inequalities must be sustained and amplified to build a more equitable and resilient Zambia for its most vulnerable citizens.

In addition, school closures resulting from COVID preventive measures meant that many children were out of school and thus had minimal supervision from school. About 4 million learners in Zambia were affected by school closures brought on 20th March 2020 for an indefinite period (23). This move resulted in the drop out of school among children from at-risk households who could not engage in online learning. According to Finn and Zadel’s survey, 89% of children attended school before the pandemic, but less than 44% were involved in online learning during the pandemic. Generally, Zambia has experienced high school dropout rates even before the COVID-19 pandemic began. For instance, the Examinations Council of Zambia recorded dropout rates of 44% among students in 2012 (25). Also, prolonged school closures when the pandemic was at its peak resulted in increased numbers of young women falling pregnant, whereas young men resorted to illicit substance use and theft [(26), p. 2]. This was partly due to increased responsibilities for supervision, primarily by women, which affected their allocation of time to multiple commitments, thereby reducing oversight for young adults.

### An overview of mental health and addiction care services in Zambia

Mental health services in Zambia have a lengthy and intricate history. In the early 20th century, mental healthcare was basic and limited to several missionary hospitals (27). Following the end of British rule, the post-independence government acknowledged the necessity of establishing psychiatric services. Consequently, Chainama Hills Hospital was inaugurated in 1966 as the country’s first public psychiatric hospital (28). During the 1990s, the country underwent a health system restructuring that disregarded mental health (29). Although there were growing demands to integrate mental health into primary care for improved accessibility, numerous obstacles impeded progress (29, 30). Several pilot programs exhibited success but lacked the required financial support and policy endorsement for further expansion. To date, mental health services in Zambia continue to confront numerous challenges Additionally, persisting stigma, inadequate mental health indicators, and limited data pose substantial barriers to service delivery, particularly within primary healthcare facilities where integration has occurred (30, 31).

In Zambia, mental health services are predominantly hospital-based and highly centralized (32). Moreover, psychotropic medications are usually costly and in limited supply at mental health facilities (10, 33) resulting in families bearing enormous out-of-pocket costs for healthcare (34). Thus, it is unsurprising that 2% of the national health budget is designated for mental health care (35). The small number of qualified psychiatrists and other mental health professionals (36), combined with an absence of public community-based substance use rehabilitation services, further complicates matters for family caregivers of young people with SUDs in Zambia.

Regarding addiction care, addressing SUDs faces multiple challenges due to a lack of resources and infrastructure (37, 38). The availability of detox and rehabilitation programs is scarce, leaving numerous individuals struggling with untreated disorders. Where services are available, treatment regimens include the prescription and administration of psychotropic drugs such as Diazepam (oral and injectable), Thiamine, Vitamin B Complex, Fluoxetine, and Niacin for those with alcohol use disorders, and Risperidone for youth with cannabis use problems presenting with psychotic features. Psychotropic medications for a standard prescription range from above K100 to more than K250. Services like counseling and psychotherapy, psychological assessments, screening for mental health conditions, linkage to other hospital services, health promotion activities in schools, and general community and youth friendly services are provided (39). In addition, faith-based and traditional healing services are also prevalent in Zambia’s mental healthcare system and are used by SUD caregivers.

However, scarce rehabilitation resources in developing countries like Zambia increase the family caregiving role (40), with the weight of SUD caregiving primarily resting on women, typically the wives, mothers, and daughters of those affected by SUDs (41). These caregivers often do not possess the necessary knowledge about SUDs or the skills to provide optimal support, leading to negative outcomes for both patients and caregivers. This situation indicates a necessity for extended therapeutic intervention and community-based support to aid the recovery process of individuals struggling with substance abuse, enabling them to maintain their recovery and alleviate the hardships faced by their caregivers.

## Materials and methods

The data presented in this paper are part of a phenomenological (Doctoral) study on the lived experiences of family caregivers of youth with substance use disorders in Zambia conducted in the pandemic context between May and August 2022 (see Supplementary File). Recruitment of study participants was done with the aid of the clinical officers at Kanyama General Hospital (KGH) Mental Health Unit, who acted as gatekeepers and provided contact details of family caregivers of young adults who attended outpatient substance use recovery services. The caregivers were contacted via phone, and details of the study were explained to them. When FCGs who were contacted refused to participate in the study, the clinical officers provided more contacts.

Participants in the study consisted of caregivers (aged 18 and above) for young adults with SUDs, such as alcohol or cannabis, ranging from 16 to 24 years old. This is because alcohol and cannabis misuse are prevalent issues among young adults in Zambia (9–13). In addition, at the global level, the World Drug Report (22) indicates that data from Western countries shows that “cannabis is a common drug of choice for young people” [(42), p. 6] citing easy availability and perceived low risk of harm as among the causes for increased use. Moreover, the latest report by the United Nations Office on Drugs and Crime (43) also shows increased cannabis use among young adults. Some scholars argue that the legalization of cannabis use for recreational purposes in most countries has led to an increase in use among most youngsters (44).

The purpose of the research was explained to potential research participants through direct phone conversations. This included discussing ethical concerns such as obtaining informed consent, ensuring confidentiality, and maintaining anonymity. Furthermore, the participants were informed about their right to take part in the study and were given the option to withdraw at any point, including during the interviews. Therefore, participation was voluntary, and the study involved a purposive sample of 30 family caregivers who gave written informed consent to participate in the research and for the interviewer to audiotape the interviews. Both male and female caregivers were invited to participate in face-to-face semi-structured interviews.

A detailed interview guide was developed by drawing insights from the key conceptual constructs of the theories guiding the main study (positioning theory and theory of caregiving dynamics) and as inspired by existing literature on the topic.

Before initiating the study, a preliminary test of the interview guide was done with individuals who shared similar traits (caregivers for young adults aged 16–24) with the intended interviewees but were not part of the study (45). This pre-testing aided in determining the clarity and comprehension of the questions and the effectiveness of the topic guides in producing data relevant to addressing the research questions (46). In general, the pilot study demonstrated that the interview guides were comprehensive and could obtain responses that would contribute toward addressing the research questions. No changes were made to the interview guide.

Two interviews were conducted, an initial interview and a follow-up interview after 4 weeks, based on the premise that collecting data over an extended period is suitable for unraveling caregivers’ experiences during the recovery trajectories of youth with SUDs, which are chronic and relapsing conditions (47). Doing this helped to capture the changes in caregivers’ experiences over time. That is, from the time their young adults were diagnosed with SUDs, to the time of the interviews. The interviews involved exploring the retrospective and current caregiving accounts of FCGs. This is because informal caregiving and recovery involve temporal phases or timelines of significant events (48, 49). All the interviews were conducted in safe spaces within Kanyama General Hospital and lasted up to 60 min.

Data was collected using an audio tape recorder by the author and transcribed verbatim with the help of two transcribers. Interviews were conducted by the author in the language that participants were fluent in (English, Nyanja, Bemba) with no need for an interpreter because the author is fluent in all the three languages. Data analysis was done inductively using the Interpretative Phenomenological Approach (IPA) proposed by Smith et al. (50). The IPA is particularly suited for examining topics that have not been extensively researched or those that involve complex or ambiguous phenomena, as well as for understanding the real-life experiences of family caregivers in their unique sociocultural context, in their own words (51, 52). With the IPA approach, interview transcripts were analyzed systematically and qualitatively on a case-by-case basis, and a narrative account was created that exhaustively presented “the researcher’s analytic interpretation” of the study’s findings, with verbatim extracts from the participants’ accounts [(50), p. 9].

Data protection of participants’ information was ensured through unique identifiers of family caregivers such as FCG1, FCG2, etc. This helped in anonymizing the caregivers and protecting their identities (Table 2).

**Table 2:** Sociodemographic characteristics of FCGs and their relationship to the young adult.

Name	Gender	Age	Marital status	Employment status	Relationship to the young adult
FCG1	Male	42	Married	Self-employed	Father
FCG2	Male	43	Married	Part-time employment	Father
FCG3	Female	41	Married	Marketeer/street vendor	Mother
FCG4	Female	43	Married	Businesswoman	Mother
FCG5	Female	38	Single	Self-employed	Mother
FCG6	Female	40	Single	Employed	Mother
FCG7	Male	51	Married	Employed (Farmworker)	Father
FCG8	Male	42	Married	Employed	Uncle
FCG9	Female	49	Widow	Employed (Maid)	Mother
FCG10	Female	54	Divorced	Unemployed	Mother
FCG11	Female	52	Widow	Hospital volunteer	Mother
FCG12	Female	53	Married	Businesswoman	Mother
FCG13	Female	46	Divorced	Trader	Mother
FCG14	Male	58	Divorced	Artisanal artist	Father
FCG15	Female	47	Separated	Teacher	Mother
FCG16	Male	48	Widower	Security guard	Father
FCG17	Female	40	Married	Hairdresser	Mother
FCG18	Female	41	Widow	Restaurant owner	Mother
FCG19	Male	49	Married	Security guard	Uncle
FCG20	Male	32	Married	Businessman	Brother
FCG21	Female	43	Married	Businesswoman (salon)	Mother
FCG22	Female	64	Widow	Self-employed	Mother
FCG23	Male	63	Married	Tailor	Father
FCG24	Female	42	Married	Unemployed	Mother
FCG25	Female	23	Married	Unemployed	Wife
FCG26	Female	60	Widow	Trader	Mother
FCG27	Female	28	Married	Trader	Sister
FCG28	Female	43	Married	Unemployed	Stepmother
FCG29	Male	37	Married	Self-employed Mechanic	Husband
FCG30	Male	58	Married	Civil servant (Office orderly)	Father

The study sample included different types of relationships between the caregivers and care recipients. Of the research participants, 17 were mothers [one mother joined her husband’s (FCG23a) interview halfway], 7 fathers, 2 uncles, 1 brother, 1 sister, 1 husband, 1 wife, and 1stepmother. These participants (males and females) were purposively selected to get a heterogenous sample of FCGs that that would provide multiple perspectives on caregiving for addicted youth. With regards the youth’s diagnosis, 13 had cannabis use disorders, 8 had alcohol use disorders, 8 were diagnosed with a combination of alcohol and cannabis use, whereas 1youth had alcohol and sniffing drugs. Overall, FCGs expressed concern about their youth having polysubstance use problems and were aware of the existence of a mixture of substances in addition to the prominent alcohol and cannabis use by the youth. The University of Zambia Humanities and Social Sciences Research Ethics Committee (HSSREC-2022-April-019) and Lingnan University provided ethical approval for the study. Institutional consent was also obtained from the Medical Superintendent at Kanyama General Hospital.

## Results

Caregivers recounted their lived experiences from the time the youth’s problem began to the time of the interview. Findings from the first and second interviews indicate that even before the COVID-19 pandemic, FCGs experienced multiple challenges, including work disruptions, financial challenges, their children’s problematic substance use and consequent dropping out of school (Table 3).

**Table 3:** Themes on the experience of addiction care before COVID-19 and during the pandemic.

Superordinate theme: the experience of addiction care	Theme	Sub-themes
Work disruptions	Missing work
	Attending to caregiving responsibilities
	Youth’s problematic and disruptive behaviors
	Young adult’s relapse
Young adult’s dropping out or school	Young adult’s preoccupation with substance abuse
Loss of income and financial difficulties	Stay-at-home orders during COVID.19 pandemic Loss of employment
Difficulties keeping the young adult indoors	Young adult’s unabated roaming and runaway tendencies Young adult’s cognitive impairments
Accessing healthcare	Adherence to COVID regulations
	Challenges in accessing medication
Forgone healthcare	Young adult’s treatment elusive tendencies Lack of family support
	Caregivers’ perceptions to a far-fetched recovery
	Manipulation and extortion of healthcare fees The young adult’s outlook on COVID
Coping mechanisms	Religious coping
	Problem-focused coping
	Emotion-focused coping

### Experience of addiction care before the pandemic

#### Work disruptions

It is worth noting that even before the outbreak of the COVID-19 pandemic, some family caregivers were laid off from work due to missing work as they attended to caregiving responsibilities for their problematic youth. Caregivers often reported work disruptions when called upon to quickly attend to the youth in instances when they became violent and exhibited disruptive behaviors or when they relapsed and needed urgent medical attention, as seen in the accounts below:

I could not go to work for fear of him creating problems around people… I was laid off at work because I was not going every day, employers are different, some are nice, and some are not nice and cannot understand my situation (FCG7, Father).

Even if I am scheduled to go to work, I fail to do so. I stay home so that I can observe him. When his temper goes down, the violence finishes, and he goes back to normal; maybe that’s when I can go to work the following day (FCG30, Father).

I am usually disturbed to find money for food, and my eyes are just on him. If I am at the market selling, I would be called often to go and see what he is doing. So, most things have been disturbed (FCG12, Mother).

The above narratives show how the youth’s problematic behaviors affected family caregivers. In times of the youth’s relapse, caregivers abandoned and missed work to monitor the young adult, and some experienced grave consequences of being laid off from work because of their erratic attendance to work responsibilities, which was affected by the need to care for their youth. For youth with runaway tendencies, their caregivers also had to forgo their small personal businesses to search for them. Some opted to stay home for fear that their child would run away.

I cannot work properly or look for money as I should…I try, I hustle, you’ll find that you plan that now let me do some work, but now being a parent and one who monitors the child, you find that one week, two weeks, she’s nowhere to be found. So, you abandon your work and get in the job of searching for her (FCG3, Mother).

He really troubled me such that I stopped leaving the house in the fear that he would run away from home…When you leave the house, you’ll hear that he ran away, and people bring him home saying we picked him from a certain place… I even stopped going to church (FCG26, Mother & Widow).

The challenges highlighted in the excerpts above show how caregivers experienced difficulties in their caregiving even before the pandemic. This was worsened during the pandemic because of the socioeconomic changes that came with it, as discussed earlier. For instance, failure to properly engage in work activities due to the constant search for a runaway youth with SUDs or the need to stay home to monitor the youth’s movements resulted in dwindled finances and opportunities to engage in other meaningful activities like attending church. The caregivers’ compromised financial situation adversely affected their capacity to care for their young people.

#### Dropping out of school

Even before the COVID-19 pandemic, most young people with substance use problems were reported to have dropped off from school, resulting from their addiction to alcohol and cannabis.

He stopped going to school on his own. We used to tell him to go to school, but he stopped. He used to be chased from school because he dressed like a junkie (FCG19, Uncle).

At first, he used to go to school to learn, but afterward, he stopped going to school and became something else (FCG30, Father).

My son used to be very brainy, and if you ask about him at his school, they will tell you about him. He stopped going to school in grade 10 (FCG18, Mother & widow).

All these demonstrate the difficulties that young adults contending with SUDs face regarding educational attainment. This disappointed family carers who experienced an ambiguous loss of “an intelligent child who would have been.” Similarly, existing research shows that students affected by SUDs faced lower academic achievement than their peers (53) because of their preoccupation with substance use (7). Family caregivers consequently had a huge burden of care in the home because their emerging adults with SUDs were not in school.

In Zambia, emerging adults transitioning from adolescence to adulthood who are affected by addiction have been highly labeled as “junkies.” This term has been normalized by citizens including FCGs, a phenomenon that has led to the entrenched stigmatization of this population. Even before the pandemic, the perceived “wasted life” of these emerging adults by members of society emanated from the youth’s persistent use of substances, compulsive use despite negative consequences, disruptions in daily life activities, and issues with interpersonal relationships. Despite the evident deleterious effects of SUDs on the youth’s psychological, emotional, physical, and social functioning, some were reported as enjoying being hooked on substances. One stepmother explained, “I asked him what is that thing that restrains you from quitting smoking? He said aah I do not know it…I cannot stop these things coz they are good” (FCG28, Stepmother). Similarly, a father of a youth with alcohol use disorder indicated that “When he has not taken alcohol, he is normal, but himself says that I do not feel good when am normal. I see people wanting to kill me, they are chasing me, and things like that” (FCG23a); depicting the unpleasant hallucinations that some individuals with SUDs experience. For such emerging adults, perpetual intoxication with alcohol and/or cannabis is better than being sober or “normal,” and the use of healthcare services comes into the picture when they are in a physical health crisis. Some caregivers indicated that their substance-dependent youth were part of a subculture of “young adult junkies” influenced by peer pressure and difficult to manage.

These children are in groups, he would like to stop but due to those groups, he still goes back… he started hanging around with the friends who smoke, and drink and he started doing those things again (FCG9, Mother & Widow).

### Impact of COVID-19 on addiction care for young adults

When the pandemic began, FCGs’ initial challenges with addiction care for their emerging adults became even more complex. This was mainly due to the loss of income and its related financial difficulties.

#### Loss of income and financial difficulties

Findings show that most FCGs worked in the informal sector as farm laborers, house helpers, salon attendants, and in personal businesses such as tailoring, mechanics, and street vending. Therefore, the stay-at-home orders for non-essential services meant losing their incomes and businesses. As such, the pandemic exacerbated the predicaments of families already susceptible to adverse economic conditions. This situation made caregiving challenging for most FCGs who experienced financial hurdles due to loss of livelihoods.

As Muzyamba (54) notes, during the pandemic, a sharp increase in unemployment and poverty levels was observed as individuals were unexpectedly cut-off from their daily sources of income and livelihoods and had dwindled out-of-pocket expenses for their survival (54).

One single mother explained how even getting a coin became a challenge during the pandemic:

During COVID-19, money was very hard to find, such that even a coin was hard to find… It was very hard to find K100 transport to take him to Chainama because I could not go alone. I needed to go with someone (FCG13, Mother).

A father who began caregiving for his son diagnosed with alcohol use disorder in 2010 indicated, “It was crucial, it was difficult… The issue of masks, sanitizers, and all… these things we usually do not have money to be buying, to make masks, buy sanitizer every time (FCG23a, Father).

The above quotes illustrate the financial difficulties experienced by carers of young adults in addiction recovery who were experiencing financial problems but needed someone to accompany them as they took their child to the country’s largest psychiatric hospital, as in the case of FCG13. Because Chainama Hospital is located on the outskirts of Lusaka City, most caregivers reported challenges with transportation, and this was worsened during the pandemic due to disrupted cash flows for most caregivers who were in personal services and self-employment. For FCG23a, he disclosed having challenges purchasing things like face masks and sanitizers during the pandemic, which were non-essentials for him before the COVID-19 outbreak.

It is worth noting that in addition to the loss of income, most FCGs lost moral authority over their youth. Carers attributed this loss to the onset of their youth’s SUDs and the consequent cognitive and behavioral changes which included increased impulsivity, disobedience, lack of self-inhibition, physical and verbal abuse. While one father noted that his son was not pleased with him, even though he was doing something good, an uncle explained his nephew’s unheeding attitude toward good advice.

He is not happy even if you try to do something good for him…he is not seeing that these people are doing good things for me… Sometimes after cautioning and encouraging him to change, he would get frustrated and keep a grudge. He would sometimes leave home and not come back again (FCG2, Father).

We started noticing his behaviour changing because he was meeting different people and his drinking habits went up. After we saw that, we tried to sit him down and talk to him, but he did not listen (FCG8, Uncle).

The experiences of these caregivers depict their diminished opportunities to communicate with, and counsel their youth. It entails a loss of caregiver control over the youth with limited moral and physical oversight.

Despite the loss of moral authority triggered by the youth’s addiction, all caregivers acknowledged that their relationship with the youth before they developed SUDs was cordial and characterized by mutual respect. This was irregardless of the socio-economic status of the FCGs. Interestingly, even after their youth’s diagnosis with SUDs, some caregivers reported that their care recipients openly expressed their love for them and acknowledged their efforts in helping them recover. In narrating the youth’s appreciation of carers’ support, FCGs gave the following accounts:

Daddy I also think about you, it’s just that you are not working, otherwise, you love me. Mummy, you take care of me, you love me (FCG23a).

Our relationship is ok even though sometimes things do not go well. Sometimes he is drunk; he does this and that, but when he is normal everything is just ok. Like the day before yesterday, I was talking to him that my son, do you know that I trust you because I know you are the one who is going to take care of me and your sister. When I told him this, he just kept quiet and listened to what I was saying without answering me. We slept, and in the morning, he went for work, and went to drink. I thought he was at the carwash he went to get drunk and came home and told me that mum I heard what you said yesterday. I know, I love my family, I love you mummy… I know that I abandoned school, but it’s not because of you, it’s me and my inappropriate behavior. But everything I am doing mummy, I’ll stop. He cried, and cried and cried, and I watched him until he finished, got up and went to sleep (FCG9, Mother & Widow).

However, despite these affirmative proclamations, the youth’s addiction presented challenges in keeping them indoors during the peak of the pandemic as they desired freedom to go out and access the substances.

#### Difficulties in keeping the young adult indoors

For the youths, the COVID pandemic did not make much difference to their situations that existed before the pandemic. They often roamed about unabated by stay-at-home measures and actively searched for cheap locally brewed alcohol and cannabis, which were readily available. Although the pandemic context required controlled movements, the young adult’s non-adherence to this increased their caregiver’s stress and anxiety.

For instance, several family caregivers reported challenges in keeping their youth indoors during the stay-at-home orders instituted by the government. The young adults continued their daily movements as they did before the pandemic, and it was difficult to constrain them. The following accounts reveal the challenges experienced by FCGs in ensuring that their young adults adhere to COVID-19 regulations:

When COVID was a problem, I used to make efforts that he should minimize his movements, but it was not possible. I was scared that if COVID was there, maybe he would be among those with COVID, or he would bring COVID home and infect his friends in the house. But it wasn’t easy because he was rarely home. When it’s morning, he will leave home. Early in the morning, he would go, and he would come maybe around 9 a.m. when he wanted to eat breakfast. When he eats, he goes, and he’ll come back at perhaps 2 or 3 p.m.; he goes again and would come back at 9 or 10 p.m. just like that (FCG9, Mother & Widow).

The child is not the kind you can make to stay home. You tell her, “look there is this disease; please stay home you rest, look put on this mask,” since the person is confused… You cannot tell her that look; there’s COVID, so do not go, do not move around; put on a face mask. She cannot comprehend that (FCG22, Mother & widow).

It was difficult because I was limited, and he would not follow COVID-19 Instructions. I reached the extent of going to buy for him cigarettes so that he did not go outside, but he still wanted to go out because he thought that the home was a jail cell until other people said that I should be letting him go sometimes (FCG12, Mother).

The above accounts of mothers of young adults with addiction problems reveal the difficulties they encountered in their attempts to constrain their children from unnecessary movements during the stay-at-home orders, what I call “quarantine-averse behaviors” that were risky and deviated from the global COVID norms that individuals were required to abide by. These mothers also had challenges having their substance-dependent children adhere to COVID-19 regulations, such as wearing a face mask. For FCG22, she attributed her daughters’ difficulties understanding the importance of abiding by the COVID-19 protective measures to a disordered mind. In the case of FCG12, her son’s non-adherence to COVID-19 guidelines prompted her to resort to purchasing cigarettes on his behalf in the quest to keep him indoors, but this proved futile because her son felt like he was being imprisoned.

Although most FCGs encountered difficulties with keeping their substance-dependent youth indoors, stay-at-home orders made the surveillance of youth with runaway tendencies much easier. These orders accorded FCGs the moral and physical oversight on their youth during the pandemic, where being cooped up was a “normalized” phenomenon. While FCGs felt they were missing out on other meaningful activities due to staying home monitoring their youth, the young adults were reported as having a sense of lost freedom.

These perceived changes in daily routines of youth with SUDs under “lockdown” caused stress in the youth who desired to go out as they did in the pre-pandemic times. In line with the Lifestyle-exposure theory (55) and the routine activity theory, risky lifestyle behaviors such as substance abuse among frequent users affects their daily routines that are characterized by a preoccupation with acquiring and using harmful substances and engagement in deviant behaviors (56). Consequently, this poses challenges in caring for this population and results in postponed healthcare because young adults are often unavailable.

These findings concur with Hawkins (57), who asserts that substance use disorders result in low levels of inhibition and poor judgment among those affected, especially emerging adults. She further notes, “The youth often believes s/he is fine, perhaps no different or even better off than peers, and may be highly resistant to any form of intervention” [(57), p. 203].

#### Accessing healthcare: adherence to COVID regulations

Although the fear of contracting the virus was widespread among FCGs and other citizens in the country (21, 54), most family caregivers in the current study braved the virus and could access healthcare professionals based on given appointments. They reported that access to care at the mental health unit of Kanyama General Hospital was facilitated by adhering to the COVID-19 regulations of washing hands with soap, social distancing, hand sanitizing, wearing a face mask in public places, and covering the mouth with an elbow when coughing and sneezing.

When we used to come here during COVID-19, we used to wash our hands and sanitize, they would give us medicine, and they would do everything quickly so that we do not waste time there (FCG17, Mother).

When we reached the hospital, they would give us face masks and they would first test us for COVID-19 (FCG30, Father).

This shows the FCGs’ willingness to respond to their young adults’ addiction care needs during the pandemic. Strict adherence to these guidelines as a pre-requisite to accessing healthcare services shows how FCGs, despite being worried about contracting the virus, did not shun away from receiving help from the hospital because they aimed to facilitate the youth’s recovery and return to normalcy.

Findings reveal that although most FCGs attended healthcare, there was a noticeable gap in treatment for young adults struggling with alcohol and cannabis use disorders that were exacerbated by the COVID-19 pandemic, where spaced appointments led to approximately 30% loss of clients who could not be retained in treatment (58). Most did not return for their scheduled appointments which were highly spaced for chronic conditions. The appointments were given for 3 months for non-acute conditions, resulting in the loss and non-retention of most young adults in addiction care.

#### Challenges in accessing medication

Regarding medication purchases, FCGs also noted challenges accessing needed medicines during the pandemic.

We would buy medicine from anywhere as long as we find it. And medicines were hard to find because when you go to a certain place, you find it’s not there, you go to another; it’s not there. So, things were challenging when the corona came… Things are difficult, borders, where things come from, are closed… where medicines come from, they say this place is closed, and people are not traveling. Borders are closed. When you come to the hospital, quite alright you can find the doctors, and they prescribe for you, but for you to find the medicine is very difficult… for us to find the medicine we used to move in different places (FCG6, Single mother).

This mother’s story demonstrates the difficulties that came with closed borders during the pandemic, particularly in accessing the needed medications to treat addiction problems. This was worse for countries like Zambia, which do not manufacture most psychotropic drugs but rely on imports from pharmaceutical companies in countries like China, India, the US, and other countries with advanced medical technologies. Although healthcare personnel were available and would write prescriptions for the caregivers when medicines were unavailable at Kanyama General Hospital, caregivers still struggled to access the medication, and this negatively impacted the recovery of their youth, making them more vulnerable to the chronicity of SUDs during the pandemic. In their phone survey during the first wave of the pandemic, Finn and Zadel (23) recorded that 16% of respondents attempted to purchase medicine, but their efforts were futile. The increase in prices and limited supply of medicines on the local market made access to essential medicines a nightmare for families of individuals affected by addiction problems.

### Factors contributing to forgone healthcare

Several factors contributed to forgone healthcare. Most of these existed before but continued and worsened during the pandemic. Issues such as the young person’s treatment-elusive and runaway tendencies, lack of financial support, perceptions of a far-fetched recovery, manipulation and extortion of healthcare fees were further exacerbated during the pandemic and resulted in forgone healthcare.

#### The young adult’s treatment-elusive tendencies

The likelihood of forgoing healthcare during the pandemic was higher for FCGs of treatment elusive youth and those with runaway tendencies. Youths who actively refuse treatment are a massive crisis in addiction care and a predictor of forgone healthcare. A study by Waldron et al. (59) mirrors the challenges parents face when they attempt to get their substance-abusing young adults to enter treatment for drug abuse. For instance, most family caregivers needed to visit the hospital with their youth. Still, they could not due to factors such as the treatment elusive tendencies of young adults with addiction problems.

In their quest for freedom from stay-at-home orders, most youth ran away from home and consequently missed out on healthcare, which their FCGs forwent because of the young adult’s unavailability. Caregivers’ narratives revealed that some youth left home to live with their substance-abusing friends, others wandered off in search of alcohol and/or drugs and made the streets their new home. One father who began caregiving for his son in 2020 remarked, “He would run away from the house sometimes and not sleep home… he was found with his friends then apart from that in 2021, he became worse. He even stopped school. He used to run away with his friends” (FCG2, Father). Similarly, a stepmother indicated, “Sometimes he would stay 2 days without coming home, after that he comes back home. When you ask him where he’s been, he says he was in the streets… it’s a challenge because he’s still in the habit of running away from home. He does not stay in one place” (FCG28, Stepmother).

Consequently, caregivers who forwent healthcare experienced adverse health outcomes for their youth, whose recovery trajectory was prolonged by a lack/limited use of healthcare. This also increased the risk of poor mental and physical health for young adults. For instance, a widowed mother who had been caregiving since 2017 to a young adult with both alcohol and cannabis use disorders and serious runaway tendencies shared the following ordeal that happened in the pandemic context:

I also went to social welfare and had him registered. I was told to take him there, but he ran away when I tried to take him. So, I went back home because I was only taking him there because they wanted to see him…I came back home and never went back to the social welfare offices (FCG18, Mother & Widow).

The above account shows the missed opportunities for addiction care for youth with runaway tendencies and the adverse consequences to their physical health. This scenario requires a strengthened community mental health system that can reach out to such a population and help reduce the treatment gap, more so in pandemic times. This is because young adults with SUDs are challenging to serve (57) and engaging and retaining them in treatment and other essential services is often problematic, resulting in poor outcomes.

Also, the spaced appointments given for people with chronic illnesses during the pandemic made most of this population fall out of care. The lack of family support also aggravated the situation of forgone healthcare experienced by carers of young people with SUDs.

#### Lack of family support

For some caregivers, a lack of support from close family members made them forgo healthcare for their youth. For instance, when FCG21 visited the hospital and did not find the healthcare professionals who had gone to attend a funeral, her husband mocked and verbally abused her for wasting transport money that he gave her and their son for travel to the hospital. She explained that:

We came here and found the grill door locked. We asked around, and we were told that the doctor had gone to a funeral… That’s how we returned home, and the father said, “You did not find them, eh? You’ve destroyed my transport, I gave you money for transport, but it’s been wasted” … we did not come back again, because we were shouted at by the one who gave us transport money (FCG21, Mother).

Later in her interview, this unsupported mother lamented that she shouldered the caregiving responsibilities unaided financially and emotionally despite having a husband. She explained that because of her husband’s negative reaction and lack of support for their son’s recovery, she did not return to the hospital until the day she was contacted for recruitment to participate in the study. Her ordeal reveals how the lack of support from significant others potentially results in forgone healthcare for young adults with substance use problems. This adversely impacts their recovery trajectories due to the lack of financial aid, impeding receiving much-needed services. As this occurred during the summit of the pandemic when access to income was a challenge for many vulnerable households, every penny counted, leading to frustrations when monies spent (in this case on transportation to the hospital) did not achieve the intended purpose. While FCG21’s husband saw it as a waste of money and refrained from financially supporting their son’s recovery, this move harmed their son’s mental and behavioral health and aggravated the chronicity of his SUDs.

Furthermore, this primary caregiver’s source of income was lost after her salon got broke. Providing holistic care to her son and his family without the support of her husband, she explained:

My son’s problem started in 2019 when he went to smoke cannabis. He has a wife and one small child at home. I am the one taking care of them. When he impregnated the lady, they brought her to us, and I am the one taking care of them…I reached a point where my salon got broke. Yes, I’ve stayed 3 months with a broke salon, because when I find money I give them for feeding, for their child, for going to the clinic, for the wife, and their house for rent (FCG21, Mother).

This mother’s loss of an independent source of income during the pandemic increased her susceptibility to her husband’s verbal abuse as she was adamant to continue providing care to a substance-abusing son, his wife, and child; a situation her husband did not approve of. She was emotionally and financially vulnerable and consequently forwent care for her son.

#### Perceptions of a far-fetched recovery

Also, frustrations stemming from perceptions of a far-fetched recovery of the youth with chronic and relapsing SUDs, which continued unabated even during the pandemic, resulted in non-take-up of care as FCGs experienced a sense of futility in their efforts to facilitate the young person’s recovery. Most caregivers were frustrated about their child’s behavior, which posed challenges in accessing and utilizing the available services. As one father remarks, “I am always worried…We think of what we will do, which seems farfetched; we are in a dilemma. How are we going to deal with it? Where will we get help, so our child returns to normal? What will we do? We have gone to many places, desiring that he should stop drinking alcohol, but we are just writing on water. Nothing is happening” (FCG23a, Father). For this father, his use of the metaphor “we are just writing on water” signals the farfetched and seemingly unattainable recovery of his son. This situation led to feelings of helplessness and an “emotional overload” (60) on the part of this male family caregiver who had run out of options and was in a predicament on the next step to take.

Similarly, in expressing her frustration and the uphill battle she was experiencing concerning her daughters’ non-recovery and the perceived lack of therapeutic gains, a widowed mother forwent healthcare after several failed attempts at facilitating the recovery of her cannabis-abusing daughter over time. For her, the daughter’s non-response to medication was a misuse of drugs that would help another person. Her unmet treatment expectations resulted in her non-continuation of purchasing medicine for her daughter from the private clinic.

I just saw that even though I was going to get her medication, I just realized that the medicine, which is supposed to help other people, I am getting, and things look like they are not changing, no let me stop. We used to come with her; when they were interviewing her in there, she would run away, and the doctors in there would chase after her and drag her back. They would jab her; maybe this injection will help but to no avail. The medicine has refused to work! Mmm, she’s just like “umungu ulya ushipya” [‘an African pumpkin that does not cook’] (FCG22, Mother & Widow).

This widowed mother’s sentiments and use of a metaphor in describing her daughters’ non-response to medication illustrate the intense frustration, disappointment, and psychosocial burdens that family caregivers experience when medical interventions for addiction problems prove ineffective. The unfortunate result is forgone healthcare for medically vulnerable youth with chronic SUDs. Even during the interview, she reported that her daughter was still in a condition of relapse. With dwindled finances during the pandemic, she could not afford to continue purchasing medicines from the private hospital for her daughter.

Since it was a private hospital, I would get the medicine, and then I realized that Ah, I will not manage. The father is not around, I am alone, and I always have to be paying money (FCG22, Mother & Widow).

Coupled with frustrations about the seemingly unattainable recovery of her daughter, FCG22 also experienced the adverse effects of a fragmented child and adolescent mental health system, contributing to her decision to abandon care. This fragmentation of mental health services preceded the pandemic but was aggravated by it. In Zambia, there is a pervasive gap in the continuum of care for adolescents and young adults with addiction concerns and other mental health problems (31, 38).

#### Manipulation and extortion of healthcare fees

Some caregivers who opted to seek addiction care services from faith-based and traditional healers forwent care when they were charged exorbitant prices and told to purchase things like anointing water, anointing oil, and other items that these healers prescribed for the treatment of the young adult, and yet saw no change as evidenced in the story below:

“When the problem was just starting, we used to go to like churches, they would pray for her by a Papa, but he never told me what exactly was causing my daughter to be the way she is… I saw that I was not finding any help because when they prayed, they needed to tell me the cause of my child’s problem, so I knew, but there was nothing like that… In the end, I gave up and told myself that I wasted time and the money I get is through struggling. When I go to a pastor, they tell me to bring such an amount of money for them to pray for my daughter’s recovery. I release that money in my poverty, they pray for my daughter, and nothing is happening. So, I just said that I’ll never go to traditional healers. If it’s traditional healers, I never tried them. If a pastor wants money, what about a traditional healer? How much will I give them? So, I just abandoned everything” (FCG3, Mother).

Also, two widowed mothers narrated the following:

I bought anointing water and anointing oil. I bought it. It was K100 that 750mls, and I was given a bottle that I should give him to drink and also to sprinkle where he sleeps. Things did not change; I stopped (FCG9, Mother & Widow).

I went to the Traditional Doctor to access medicines, and we were told that he needed to be cleansed of ghosts, and he requested K1500, but I failed to find it (FCG26, Mother & Widow).

These mothers’ accounts show how some FCGs who sought help from faith-based healers and traditional healers had to forgo healthcare because of logistical constraints like lack of finances and a perceived lack of positive outcomes for the young adult after accessing treatment. Also, we see how sociocultural factors such as beliefs on the causes of addiction (for example, ghosts) and other mental illnesses shape mental health care in Zambia (61). Although accessing healthcare services from traditional and faith-based healers is predominant in Zambia, some providers extorted their service fees during the pandemic, resulting in the abandonment of care among family caregivers.

#### The young adult’s outlook on COVID

It is worth highlighting that forgone healthcare was also due to a perceived lack of susceptibility to contracting the virus among young addicts. Such views made some adolescents and young adults not attend healthcare during the pandemic. Moreover, before accessing any services at the hospital, everyone needed to get tested for COVID-19. As Daabek and colleagues note, in addition to socioeconomic status, “personal conducts and/or beliefs, can lead individuals to forgo or postpone identified healthcare needs” [(62), p. 2,973]. This was particularly true for most young adults with addiction problems who believed that COVID was a scam, and they were not among those who would contract the virus:

During the time of COVID-19, he never used to pay attention to it. He used to refuse that there’s no such a disease, but for us, we used to adhere (FCG28, Stepmother).

Even when you talk, he would say COVID is a scam. I am just ok. I cannot get sick, others will get sick (FCG9, Mother & widow).

Contrary to such beliefs held by these young adults, studies have shown that individuals with SUDs are at a higher risk of COVID-19 infection because SUDs are associated with various cardiovascular and pulmonary diseases (63, 64). This population’s multiple comorbidities make them highly vulnerable to infections, which are risk factors for COVID-19 (65). This demonstrates that forgoing healthcare for this population during the pandemic has potentially deleterious effects on their health into adulthood.

In highlighting the difficulties encountered in having the youth adhere to COVID regulations, one uncle explained, “My nephew used to throw away the mask if he did not feel like having it on” (FCG19, Uncle). This demonstrates the increased risk of contracting the virus in this population of emerging adults who are not in their right frame of mind because of an impaired cognitive capacity emanating from chronic addiction.

Despite the various challenges and reasons for forgoing healthcare, most family caregivers adapted to their situation and used different coping strategies based on their caregiving context.

### Coping mechanisms employed by family caregivers

Prior studies have shown that caregivers frequently use coping strategies to navigate the terrain of providing care to those with addiction problems. Family caregivers have had to navigate a pandemic while also managing the addiction of their loved ones. During COVID-19, these caregivers faced additional stressors, such as social isolation, economic hardships, and inadequate access to support resources, as seen in the current study.

While seeking support from online communities, reducing stress through self-care activities such as meditation and exercise, and utilizing virtual therapy sessions were commonplace among FCGs in developed countries (66), FCGs in the current study coped with their challenges in diverse ways and employed psychological and emotion-focused coping, as well as problem-focused coping strategies, albeit in a unique way. Although several family caregivers used ingenious coping strategies that helped them adapt to their situation and the crisis, some strategies were maladaptive and had adverse long-term effects.

#### Religious coping

Religious coping was common among FCGs, who all subscribed to Christianity, albeit with different religious denominations. They reported having an inner resilience based on their belief in God and that whenever things became very tough in their caregiving, they left everything to God through praying. This staunch religious belief can be seen in the expressions of FCGs below:

I’ve seen that the burden is lighter; it’s because every time of my life, I have put myself in God… Yes, I am really strong in prayers. Even for this child to be alive today and they have not killed her, maybe it’s being strong in prayers (FCG22, Mother & Widow).

Do you know that taking care of such a person is not easy because the landlord can even chase you out of the house and sometimes the neighbors laugh at me that I have a crazy child, so I just need to be strong, that is why I have said I also need to pray, even Jesus said that we need to be praying. So, we pass through different problems and leave them in God’s hands (FCG7, Father).

#### Emotion-focused coping

Concerning the experience of stigmatizing sentiments from some community members, caregivers found ways of managing the stigma attached to having a substance-dependent child. Using emotional coping, they often ignored stigmatizing comments and pretended not to have heard the negative sentiments. Some quickly entered their homes and started praying, crying to God, or reading the Bible, whereas a few directly confronted the perpetrators by giving them a piece of their mind through real-life examples, as FCG21 did when she said:

Being lame comes even in adulthood. You can be born without any defect, but you may develop problems with your leg, so let us not laugh at each other, those of us who have borne children. I never knew my son would find himself on such a path (FCG21, Mother).

By retorting in this manner and confronting societal stigma, this mother positioned herself as her son’s advocate and, at the same time, conveyed to the perpetrators of the stigma that every young adult is at risk of addiction problems and that parents should desist from stigmatizing tendencies.

Conversely, a father reacted to stigmatizing sentiments by posing thought-provoking questions to perpetrators:

Have you ever thought if it was your son or daughter who’s in the same situation, how would you take it? Then they would say mm I think that’s a very good idea, so we should not be laughing at such people, but we should just find ways, if there’s a way of helping, let us help (FCG1, Father).

Regardless of their psychological and emotional challenges, family caregivers noted positive changes in their daily lives. By reframing their minds, they developed positive self-perceptions of growth and gained new insight into how to care for a substance-dependent young adult.

“I see myself as a grown-up person… I just feel that I am a person who is responsible” (FCG6, Single mother).

“Now I have experience in taking care of him. I do not have to speak to him rudely. When giving him food, I do not have to call him harshly. I now have experience, and I know him” (FCG26, Mother & Widow).

#### Problem-focused coping

With regard to problem-solving coping mechanisms, some family caregivers engaged in a re-ordering of their priorities for caregiving. For instance, when medications were scarce and expensive due to border closures, the first thing that FCG6, a single mother, did was to make sure that she bought the son’s medicine to last him a month before anything else. Although doing this was less financially stressful for her, it posed challenges in meeting the needs of her other children, like buying enough food for them.

When I have money, I buy medicine for a month. Now if I do not have money? And we make sure that before we even think about food, we have to think about his medicine and how long it will last…if I get money and buy food, where will I get the money to buy his medicine? So, you find that we reduce on other things. I have to make sure I buy medicine that will last him a month (FCG6, Single mother).

This single mothers’ experience illustrates how families of substance-dependent youth often forgo other daily life necessities like buying enough food in their quest to meet the medication and health needs of the child in recovery from addiction. The situation is dire for families experiencing financial hurdles like this lone mother’s.

FCG21, a mother who was also experiencing financial challenges after her salon got broke due to caregiving responsibilities and poor business during the pandemic, got a loan to help her solve her financial problems as well as support her cannabis-abusing son, his wife, and child all of whom were under her custody. Although getting a loan was a short-term measure to cushion the financial difficulties, the burden of care was huge. It required a sustainable source of income, which many FCGs desired during the pre-pandemic and more so during the pandemic.

In alleviating their financial hardships, most FCGs leveraged the support of close family members such as other children. For instance, FCG3 engaged her daughter in selling merchandize (street vending) since their father was diagnosed with a heart condition and could not do taxing work. The primary responsibility fell on this female caregiver, who had to negotiate to be a mother, caregiver, and breadwinner for the entire family.

Some caregivers opted to end the manipulation and extortion by traditional and faith-based healers who wanted to make more monies during the pandemic by refraining from accessing such services (see FCG3’s story on page 21).

To cope with the pandemic’s challenge on household feeding and accommodation expenses, some affected families reduced the usual three meals per day to only one meal and resorted to finding much cheaper rented houses regardless of family size. As FCG8 notes, “During COVID-19, at work, they told us to stop going for a while. When we stopped working, we had challenges at home because we started eating once daily, around 18 h, and the young children would be problematic. So, we would find some foods like rice for them. The other issue was on rentals. We stopped managing to pay for rentals. We would stay 4 months without paying until we find some affordable apartment” (FCG8, Uncle). This account illustrates the precarious experiences that FCGs had when they were put on hold at work as a pandemic measure. They had to adjust their feeding and living arrangements to survive, revealing the shifting priorities that FCGs had during the summit of the COVID-19 pandemic.

Conversely, caregivers of youth with violent tendencies resorted to working on a shift basis to monitor the young adult better. FGC16, a widower and security guard, decided to change shifts because he feared that his cannabis-abusing son would harm his siblings when left unsupervised for a long time. He did this to ensure the safety of his younger children, who were often left home alone after the demise of their mother.

He started becoming so violent, and he could even break all properties…since I am a single parent… It was challenging for me to take care of him because the moment I would leave him alone in that house, he could start beating the other children. So, I said, since I work at night, he might kill one of the…siblings (FCG16, Father & widower).

Despite having this strategy to cope with his situation, this widowed caregiver acknowledged that balancing work requirements with family caregiving was a highly stressful experience for him, and he wished that his wife was around.

A mother who also had safety concerns for her son with runaway tendencies devised a strategy to keep the son safe and reduce worrying about his whereabouts. FCG26 sent the son to her sister-in-law, whose house was in a wall fence, to prevent him from leaving home during the pandemic. She also did this to avert the stigmatizing sentiments of people in her neighborhood, who constantly insulted and mocked her son whenever he went to their yards.

I was not free when he would trouble me a lot with his constant movements, he would go to other people’s yards, and they would hurl insults and chase him, saying, go to your mother, you mad person, do not bring your madness here… So, there was a time I took him to my sister-in-law, where there is a wall fence so that he could stay there (FCG26, Mother & Widow).

Despite taking this move, her coping strategy was short-lived, as she disclosed later in her interview that “he stayed for a while and started troubling that he wants to come back home.” This shows how disorienting caregiving for an emerging adult with addiction problems could be due to their unpredictable behaviors, a finding that has been widely cited in the extant literature on caregiving for adolescents and family members with substance use problems (59, 67, 68).

Similarly, to cope with his son’s uncontrollable movements, a father sent his alcohol-abusing son to the village because he thought it was much safer in terms of low COVID levels and that the young person would not contract COVID even if they moved around as opposed to roaming the city where cases were high. His decision was with the view that the youth would come back to Lusaka after the pandemic had receded.

Furthermore, to keep his out-of-school and not-in-employment youth busy, one male caregiver engaged his son in agricultural activities to divert his focus from searching for and abusing substances. Doing this worked well for FCG30 because his son was interested in farming.

However, it is worth noting that although FCGs in this study employed ingenious coping strategies based on their complex situations, some problem-focused coping strategies were to the detriment of family members supporting the caregiver. For instance, one widowed mother (FCG9) engaged the daughter to work on her behalf as a maid so that she could focus on her caregiving responsibilities for her son with cannabis use problems. Unfortunately, this approach resulted in her daughter dropping out of school. This FCG inadvertently had two children out of school due to addiction in the family. As Mikulić and associates (69) note, addiction care is a family burden that “affects many aspects of family members’ lives” [(69), p. 2], in this case, the educational prospects of FCG9’s daughter.

## Discussion

Forgone healthcare is a critical issue that affects millions of people worldwide. It refers to the healthcare services people do not utilize or access despite the need, for various reasons including cost, accessibility, and personal beliefs. The literature shows that forgone healthcare is prevalent among vulnerable individuals with chronic conditions (16, 70), including people with addiction problems (71). Findings in the current study reveal how routine care for young adults with SUDs was hampered by structural and personal factors, resulting in an underuse of SUD recovery services in this group. For instance, there was a considerable change in the financial situation of most FCGs during the pandemic, and this was for the worst, where they experienced an unprecedented deterioration in their incomes. Even before the pandemic, the economic challenges and huge out-of-pocket bills that most family caregivers had resulted in foregone healthcare due to a lack of transportation fees and funds to purchase expensive psychotropic drugs. During the pandemic, some pharmacists extorted prices due to limited supply and increased demand, challenging medication access.

Research conducted by Haley and colleagues in Spring 2021 (72) shows that parental caregivers with low incomes experienced increased difficulties in affording the required care during the pandemic than those with higher incomes. These authors argue that “Challenges accessing and affording health care among parents with low incomes could compound the other hardships they were experiencing before the pandemic and that were likely exacerbated by the crisis” [(72), p. 2], a scenario similar to that of carers in Zambia. This infers that parental income was affected during the pandemic leading to forgone healthcare for many. Similarly, forgone healthcare was reported in the US in the initial phase of the pandemic (73). Financial concerns and the fear of contracting the virus were among the reasons for foregoing care resulting in missed administrations of prescribed medications.

Contrary to the findings in the current study, Markoulakis et al. (66) found that caregivers of youth with mental health and addiction problems in Canada forwent care due to the perceived unavailability of services and as a way to keep their young person out of risk through not allowing them to visit the hospital. Their findings reveal differences in caregivers’ experiences between those in developed countries and those in LMICs like Zambia. For instance, FCGs of youth with SUDs in Zambia, despite facing the imminent risk of contracting the virus, sought addiction care for their adolescents and young adults by following the requirements for accessing mental healthcare during the pandemic. Despite these differences in experiences, financial constraints due to job losses were reported in the study done by Markoulakis and colleagues and in the current study, posing a challenge in the capacity of these family carers to support their young adults. This illustrates the cross-cutting and perversive impact of financial hurdles on the treatment and care of youth with addiction problems in the pandemic context, resulting in forgone healthcare for some.

The poor addiction healthcare help-seeking behaviors and perceptions, particularly among young adults with treatment-elusive and runaway tendencies, compounded caregivers’ problems, created a constant burden for the family caregivers, and resulted in forgone healthcare. Pham et al. (74) also established forgone healthcare among in-school and out-of-school adolescents with mental health concerns in Indonesia. For those in school, perceptions of their problems resolving with time, and the fear of doctors’ reports made them forgo healthcare, whereas those out-of-school were affected by accessibility factors such as the cost of care and lack of transport, and limited knowledge on available services. Their findings on what influenced forgone healthcare among young adults depart from those in the current study where most youth forwent care due to poor cognition emanating from addiction, and a lowered sense for the need for healthcare among treatment-elusive youth with chronic SUDs.

This scenario resulted in family caregiver’s perceived far-fetched recovery of the youth and increased their frustrations about the futility of their efforts when the young person relapsed. Consequently, FCGs ditched help-seeking on behalf of their young adult. Also, the highly fragmented child and adolescent mental health system and poor continuum of mental health care in Zambia exacerbated forgone healthcare for this population. Most young adults with SUDs fell through the cracks of a fragile and uncoordinated mental health care system. These barriers to care and their impact on forgone healthcare were also established by Tsuzaki and Taira (75) among medicare beneficiaries with non-COVID related illnesses. For them, factors such as availability of appointments for telehealth care, COVID-19 vaccination status, region, race, age, sex, and ethnicity influenced forgone care during the pandemic. Their findings reveal that forgone healthcare is influenced by different factors in different contexts, signaling the importance of context-specific interventions.

Taken together, reviewed studies on forgone healthcare all show financial challenges as a significant barrier to care during the pandemic. With the loss of work during the COVID-19 pandemic, most families missed or postponed care due to costs (76).

Regarding the lived experiences of family caregivers given herein, this paper argues that the COVID-19 pandemic deepened the old constraints and brought new challenges for FCGs of emerging adults grappling with addiction. It is noteworthy that the caregiver burden existed before the pandemic in terms of financial challenges, hopelessness, work disruptions, and loss of jobs due to missing work to attend to caregiving responsibilities. However, as noted earlier, the loss of employment was worsened by the COVID pandemic due to stay-at-home orders and the closure of businesses. Since most FCGs are in the informal sector, they were gravely affected. Therefore, as with a number of studies on forgone healthcare, findings of the current study confirm that the COVID pandemic exacerbated the initial challenges experienced by FCGs, more so carers of young addicts.

Although the COVID-19 crisis is over and we are in a (post) pandemic era, the caregiving burdens of these vulnerable family carers, pre-existed the pandemic, are likely to continue and have been exacerbated by the pandemic. This is because of the unabated structural and personal impediments to mental healthcare in Zambia. As in the past, minimal support continues to exist for FCGs of youth with addiction problems and other mental disorders due to a poor understanding of mental health problems (31, 32, 38), the highly stigmatized nature of addiction and the perceptions of addiction as a moral failing (77). The study revealed that most parents and guardians need financial, emotional, instrumental, informational, and respite support. In addition, the Zambian economy is still experiencing the adverse effects of the pandemic, and economic recovery is at a snail’s pace. For most FCGs whose livelihoods were affected during the peak of COVID-19, their financial situation remains critical even in the (post) pandemic era, making access to needed healthcare services and treatment regimens an ongoing challenge.

Young adults with chronic untreated SUDs continue absconding from home, engage in risky behaviors such as heavy abuse of alcohol, cannabis, and other concoctions of harmful drugs, and remain out of school and employment, creating more burdens for their FCGs. The easy availability, accessibility, and affordability of harmful substances in Zambia (10) depicts the unaltered and continued use of substances. This contradicts European scholars’ assertions, “We may expect decreased levels of substance use in the short term due to decreased availability and affordability.” [(78), p. 2]. While this holds for most developed countries where low availability, skyrocketing prices, and financial impediments associated with the pandemic resulted in the decreased use of substances, the Zambian scenario offers a different perspective and extends our understanding of changing patterns of substance use in pre, during, and post-pandemic times.

Interestingly, most young adults in the current study were unaffected by school closures resulting from pandemic measures. This is because they were already out of school before the pandemic. Moreover, their dropping out of school was attributed to the abuse of alcohol and cannabis. Being out of school during the pandemic meant the burden of care was more for FCGs, who had to juggle caring for their young adult in the home and dealing with disruptive behaviors and other responsibilities. This shows that the pandemic was not responsible for the change in educational attainment for young adults who were not in school before the onset of the COVID-19 pandemic. Moreover, even during the May to August 2022 interviews, most FCGs reported that their child was not in school.

While both male and female caregivers had similar experiences of caregiver burden, emotional distress, work disruptions, and financial challenges, nuanced differences were also evident in these carers’ experiences. For instance, lone female caregivers who were single, divorced, widowed, and those with estranged partners desired more physical and financial support. In contrast male caregivers wanted instrumental support with activities of daily living. Concerning SUD stigma, male FCGs responded to stigmatizing sentiments from community members with food for thought questions to the perpetrators, whereas female FCGs used more of emotional statements justifying that they never envisaged their children in such a predicament of having SUDs (see page 24). Regarding decision-making in the healthcare setting and their relationships with healthcare professionals, male caregivers were more assertive than female caregivers. Males actively engaged the healthcare personnel, asked critical questions about the youth’s diagnosis and care, whereas most females often heeded to practitioners’ advice as experts. This finding reflects the sociocultural context of the study where males are expected to take lead in decision making while women assume the listening role. In the same vein, when dealing with their youth’s problematic behaviors, male FCGs were restrictive and expressed authoritarian attributes as opposed to female FCGs who were more nurturing. For example, male carers used physical restraints like chaining their youth with runaway tendencies to curtail their movements and keep them indoors.

Although family caregivers experienced multiple challenges, they also found ways of coping. Problem-focused coping was the most predominant among them. As earlier noted, perceptions of a far-fetched recovery made some FCGs forgo healthcare at some point. Despite this, FCGs were still actively searching for help from different sources on behalf of young adults, such as faith healers, spiritualists, traditional healers, and herbalists. The aim was to secure the recovery and return to normalcy of the affected young adults.

Overall, steps were implemented to assess the quality of the primary data, encompassing procedures to ensure and uphold the trustworthiness of the qualitative findings presented in this paper (79). This procedure included peer reviews conducted by supervisors in the quality control procedures. These reviews ensured the appropriateness of the study by providing feedback on various aspects such as literature review, data collection methods, data management processes, data analysis procedures, and research findings. Member checking was utilized to ensure rigor and establish the credibility of the research (80). Before the second interview, caregivers could review the transcripts and confirm if the transcribed content accurately reflected their interview discussions. Following the second interview, transcripts were shared with participants with access to working emails and smartphones for further verification. Coherence was further ensured by re-listening the recorded interviews and comparing them with the transcripts. Furthermore, sensitivity to the study context was considered throughout the study process. Having the lived experience as a Zambian national, and the adherence to the values and norms of Zambian society helped design the study and my positionality and cultural sensitivity as a researcher.

### Limitations

Since the study’s findings are based on the lived experiences of a small purposive sample of FCGs of adolescents and young adults with SUDs from Kanyama General Hospital’s Mental Health Unit, the results cannot be generalized. The carers’ experiences in this study may differ from those in other settings and caregivers not attending outpatient SUD recovery services from KGH. However, this study provides an in-depth understanding of FCG’s experiences and the meanings they attach to them. Since the research was conducted in an urban setting in Zambia’s capital, Lusaka, there is a need for future studies to consider rural FCGs. A longitudinal study over a longer period would also aid in gaining detailed insights into the experience of FCGs of young people with addiction problems over an extended period, particularly in LMICs, which lack such studies.

Being a study that involved in-depth face-to-face semi-structured interviews, the possibility of response bias due to my presence as the interviewer was inevitable. As Thomas et al. (81) note, the existence of an observer (such as a researcher) causes narrators to present altered accounts of their experiences compared to the anonymity provided by online storytelling. With this, future studies can also consider the alternative of getting the lived experiences of FCGs through online narratives.

## Implications for policy and practice

The study findings presented herein have implications for SUD caregiving, youth mental health legislation, policy, and services. Regarding the work disruptions encountered by FCGs in this study, there is an urgent need for flexible leave programs for FCGs employed in the formal sector, enabling them to prioritize caregiving without worrying about missed workdays. Since many FCGs were engaged in informal employment, there is a need for targeted social protection policies and interventions to ensure their well-being. For instance, integrating them into the Social Cash Transfer program as beneficiaries would alleviate the financial challenges experienced by these caregivers.

The caregivers’ narratives also reveal a necessity to enhance the accessibility of evidence-based care and assistance services for caregivers and their young adults. This involves expanding the availability and affordability of substance abuse treatment centers. In addition, the manipulation and extortion experienced by caregivers call for developing legislation to govern the practices of traditional and faith-based healing practitioners, who are among the key service providers in the Zambian mental health system.

Furthermore, the logistical and in-person barriers to healthcare access experienced by FCGs calls for the consideration of telehealth in SUD recovery services in Zambia. Moreover, telehealth treatment is seen as the “new normal” in addiction care worldwide, where outpatient care is envisaged as hybrid. That is the use of telehealth and in-person services. During the COVID-19 pandemic, telemedicine has been seen to minimize the risk of virus transmission while eliminating various logistical obstacles tied to in-person treatment services (82).

While most countries especially developed countries have responded to challenges in addiction care during the pandemic using telehealth and other robust approaches, Zambia is yet to implement comprehensive telehealth services (83) for SUD recovery services for young adults and their family caregivers. A Lack of virtual care for treatment-elusive youth with chronic SUDs poses a double burden for FCGs. Often, carers have challenges engaging their young adult in in-person outpatient services. In developing nations like Zambia, the scarcity of rehabilitation resources leads to the chronicity of SUDs and, consequently, a higher caregiving burden.

It is worth noting that individuals contending with addiction problems are high-risk groups that need workable interventions to support their recovery journeys in the post-COVID era.

Given the unpredictable pandemic, including those that will emerge, SUD recovery services for young adults in LMICs need to urgently adapt innovative technologies like telehealth and other locally feasible approaches (84) to avert disruptions to the continuity of addiction care (82) brought about by the reported treatment-elusive tendencies of young adults with SUDs. Doing this will fill the gap when young people and their families cannot attend face-to-face consultations. Due to their high vulnerability socially, economically, and health-wise (78), there is a need for tailor-made addiction care plans that are context-specific in the post-pandemic era for emerging adults grappling with substance use disorders.

The impact of forgone healthcare on individuals cannot be overstated, as it can lead to severe health complications, decreased quality of life, and even mortality. Additionally, society may experience lower productivity, higher healthcare costs, and reduced economic activity due to the inability of individuals to receive healthcare services.

Furthermore, putting off care for young adults grappling with SUDs has adverse outcomes for their recovery trajectory. More coordination of services among different providers (public, private, traditional medicine, and faith-based healing) is urgently needed to deal with the fragmentation of child and adolescent mental health services and challenges in the continuity of care.

To minimize forgone healthcare, particularly in a pandemic context and beyond, the Zambian government and other key stakeholders should develop robust mental health services and support for affected family caregivers and care recipients. This may involve implementing policies to reduce healthcare costs and increase mental healthcare funding and supply of psychotropic medications. Reducing healthcare costs through measures such as price transparency and negotiated drug prices would be an approach that might cushion the impact of out-of-pocket payments borne by family caregivers. Healthcare providers should offer more accessible care options by strengthening community mental health services and bringing them closer to the people. Telemedicine and other innovative technologies may also help to reduce forgone healthcare. This will help address the problem of relapse and the gap between healthcare institutions and the community.

To tackle the stigma experienced by FCGs, the government and critical stakeholders should implement awareness programs to educate and sensitize community members on mental health problems, including addiction. Doing this will improve the mental health literacy of the population and reduce stigma toward persons and families affected by addiction, commonly known as a family disease (69). Also, the Zambian government should consider mass recruitment of mental health social workers in public health facilities, who will act as a bridge between families in the communities and healthcare facilities. Follow-up for treatment-elusive youth will be much easier when social workers are engaged as healthcare facilitators for this medically vulnerable population.

In addition, the experiences of family caregivers necessitate their inclusion in the policy formulation process. This is because FCGs’ voices are largely missing in policymaking in Zambia. Including them would lead to the creation of policies that address the actual and not perceived needs of caregivers of young adults with SUDs.

Also cardinal, and a much more feasible and less expensive approach is for the creation of spaces (in healthcare facilities and the community) where caregivers can share their lived experiences and encourage one another. Creating such platforms for caregivers would reduce feelings of isolation and stigma and at the same time lead to more SUD caregivers who are often a hard-to-reach population being reached by available services thereby mitigating forgone healthcare. This is because although several caregivers acknowledged that there were other FCGs in their situation due to the prevalent SUDs among youth in the communities, none of them outrightly suggested having a community of caregivers to share notes. This might be due to internalized stigma and the marginalization of FCGs of persons suffering from addiction in Zambia, something that is considered as a personal misfortune. Although this was the case, most FCGs made recommendations for government support to other caregivers like themselves especially with the need to regulate and curb the sale of harmful substances that were reportedly cheap and easily accessible to the youth.

## Conclusion

Based on the lived experiences of family caregivers of emerging adults grappling with addiction, the present study shows why FCGs forwent care for this medically vulnerable group. Forgone healthcare was due to factors such as treatment-elusive tendencies among some young people with SUDs, lack of family support, financial challenges, FCGs’ perceptions of a far-fetched recovery, manipulation and extortion of healthcare fees by some traditional and faith healers, challenges in medication access due to border closures during the pandemic, and the youth’s negative outlook on COVID, perceptions of being unsusceptible to the virus and the consequent non-adherence to health interventions.

It is worth highlighting that forgone healthcare before, during a crisis, and beyond is a complex problem requiring a multifaceted approach. By expanding insurance coverage to cater to those in the informal sector (like most FCGs), reducing healthcare costs, and improving healthcare access, policymakers can ensure that patients and their families receive the care they need when they need it without incurring undue financial burdens or other negative consequences. Therefore, the current study’s findings highlight the need for continued support for FCGs of young adults struggling with addiction in Zambia and other countries with similar socioeconomic contexts.

### Strengths of the study

Among the strengths of this study is the sample representativeness in terms of the inclusion of male caregivers, an uncommon phenomenon in most studies on addiction caregiving for young adults in Sub-Saharan Africa. A plethora of studies only capture the experiences of mothers. This study had a total of 11 male caregivers which included fathers, uncles, a brother, and a husband. Having this representation of the male caregivers in a patriarchal society like Zambia, where family caregiving is highly perceived as a preserve of women is one of the strengths of the study, which revealed nuances of addiction care for young adults with problematic substance use.

The longitudinal design of the study (albeit short-term due to the nature of the main study) where two interviews were conducted in a space of 4 weeks provides valuable insights into the long-term effects of SUD caregiving on family caregivers. In addition, getting the retrospective and current experiences offers a holistic understanding of FCGs’ experiences over time.

The study’s policy and practical implications are also a strength of the study, including the study context where studies on forgone healthcare are limited. For example, findings on the manipulation and extortion of FCGs by traditional and religious healers as influencing forgone healthcare add to the addiction caregiving literature. These findings depict the importance of understanding the caregiving context and its differential impact on the lived experiences of FCGs thereby resulting in a comprehensive analysis of forgone healthcare.

## Data availability statement

The datasets presented in this article are not readily available because the datasets in this qualitative study cannot be shared in their original form due to confidentiality concerns. Requests to access the datasets should be directed to IK, ireenkangwa1@gmail.com.

## Ethics statement

The studies involving humans were approved by Humanities and Social Sciences Research Ethics Committee (HSSREC) of the University of Zambia and Lingnan University. The studies were conducted in accordance with the local legislation and institutional requirements. The participants provided their written informed consent to participate in this study.

## Author contributions

IK carried out all aspects of the study, from design, data collection, analysis, and interpretation of findings, and drafted and submitted the manuscript.
