# Supplementary material for: Forgone healthcare for medically vulnerable groups during the pandemic era: experiences of family caregivers of young adults with substance use disorders in Zambia
Source: Front Public Health. 2024 Mar 8;12:1250608. doi: 10.3389/fpubh.2024.1250608 (PMC10959020; doi:10.3389/fpubh.2024.1250608)
Supplement: Supplementary file 1 [file Data_Sheet_1.docx]

**Supplementary file (excerpt of main interview guide containing questions specific to the topic of the article)**

**Youth’s behavioural and cognitive changes overtime**

6. Please tell me about the behavioural and mental changes that the youth has been experiencing from the time he/she was diagnosed with SUDs.

- How have you dealt with these changes?

- Have these changes been difficult to manage?

- Can you describe how these changes affect how you provide care to the youth?

**Experience of healthcare service use (public services)**

7. After the youth was confirmed as having a substance use disorder, what were your expectations for the youth’s care?

8. What are your experiences of healthcare service use for the youth? (Please talk about physical and mental health service use)

-Getting help

-Relationship with healthcare professionals

-Communication with healthcare professionals

-Decision making, trust in healthcare professionals

9. What are your expectations for the youth’s care now?

**Experience of using faith-based healthcare services**

7.After the youth was confirmed as having a substance use disorder, what were your expectations for the youth’s care?

8.What are your experiences of healthcare service use for the youth? (Please talk about physical and mental health service use)

-Getting help

-Relationship with healthcare professionals

-Communication with healthcare professionals

-Decision making, trust in healthcare professionals

9. What are your expectations for the youth’s care now?

**Experience of using other alternative treatments/services**

10. What made you choose these treatment options?

-How different are they from other treatment services?

11. I’ve heard some people say that ‘family caregivers of young people with substance use disorders experience stigma/are stigmatized’, what do you think of that?

-How do you deal with stigma/stigmatizing behaviours from others?

**COVID-19 and access to and utilization of healthcare services**

12. Please tell me about your experiences of healthcare service use for the youth during the COVID-19 pandemic.

- Is there something you would like to say before we end this interview?
